# Supplementary material for: When Two-Fold Is Not Enough: Quantifying Uncertainty in Low-Copy qPCR
Source: Int J Mol Sci. 2025 Aug 12;26(16):7796. doi: 10.3390/ijms26167796 (PMC12387016; doi:10.3390/ijms26167796)
Supplement: Supplementary file 1 [file ijms-26-07796-s001.zip › Supplementary Figures_v.pdf]

Supporting Data and Extended Methodological Details for qPCR Variability and Multiplex Performance

1. Limits of detection (LoD) *(Figure S1)*

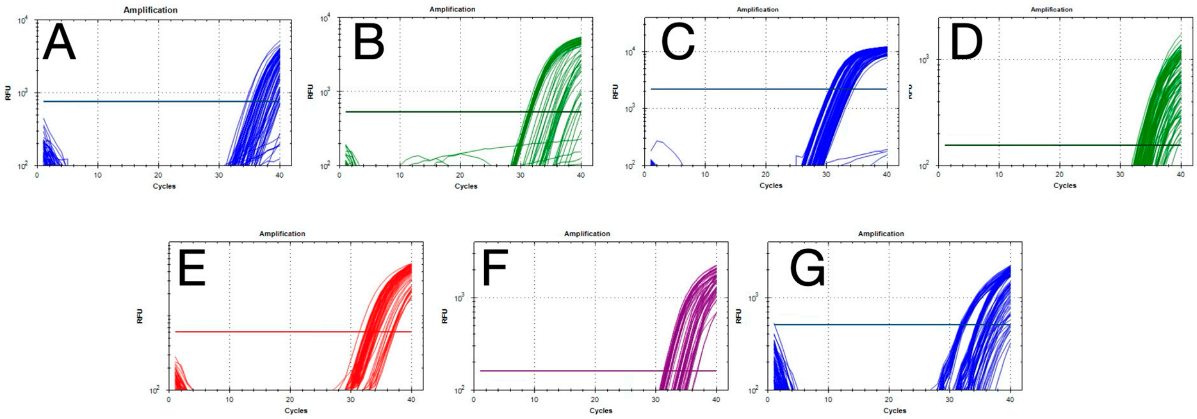

H

Copy no	50		20		5		LOD
n=24	+ves	% +ve	+ves	% +ve	+ves	% +ve	
GAPDH-A	24	100	23	96	8	33	**20**
GAPDH-B	24	100	19	76	5	21	**50**
ACTA2-A	24	100	20	83	4	17	**50**
ACTA2-B	24	100	20	100	19	79	**20**
CDKN1	24	100	24	100	15	63	**20**
IGF-1	24	100	24	100	15	63	**20**
C. auris	24	100	24	100	23	96	**20**

Supplementary Figure S1. Limits of detection established for the seven assays used in this study. A replicate was recorded as positive if it produced a detectable Cq value. Limit of detection, LoD, was defined as the lowest template concentration at which all 24 replicates were detected. Amplification plots for serial dilutions (50, 20 and 5 copies/reaction) are shown. **A.** GAPDH-A (FAM) **B.** ACTA2-A (HEX). **C.** GAPDH-B (FAM). **D.** ACTA2-B (HEX). **E.** CDKN (TxR) **F.** IGF-1 (Cy5). **G.** *C. auris*. **H.** Summary table showing the number of replicates (out of 24) yielding a detectable Cq value at each input level. Detection rates are expressed as % positive. All Cq values are listed in the supplementary data file.

2. Pipetting accuracy and small reaction volumes *(Figure S2)*

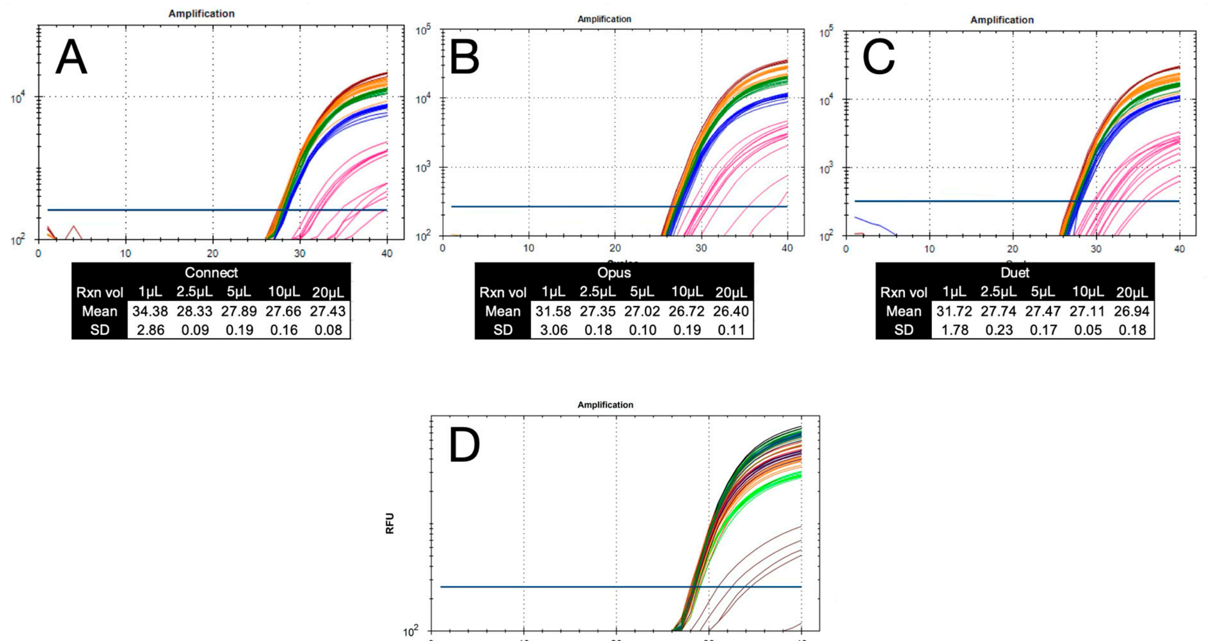

Connect					
Rxn vol	1µL	2.5µL	5µL	10µL	20µL
Mean	34.38	28.33	27.89	27.66	27.43
SD	2.86	0.09	0.19	0.16	0.08

Opus					
Rxn vol	1µL	2.5µL	5µL	10µL	20µL
Mean	31.58	27.35	27.02	26.72	26.40
SD	3.06	0.18	0.10	0.19	0.11

Duet					
Rxn vol	1µL	2.5µL	5µL	10µL	20µL
Mean	31.72	27.74	27.47	27.11	26.94
SD	1.78	0.23	0.17	0.05	0.18

Rxn vol	1µL	2µL	3µL	4µL	5µL	6µL	8µL	10µL	12µL
Mean	32.93	28.63	28.54	28.38	28.32	28.23	28.26	28.25	28.13
SD	1.56	0.18	0.17	0.18	0.31	0.10	0.10	0.10	0.05

Supplementary Figure S2. Effect of reaction volume on Cq values using low-copy GAPDH-A template on three block-based instruments. Reaction volumes of 1, 2.5, 5, 10, and 20 µL were tested on (**A**) CFX Connect, (**B**) CFX Opus, and (**C**) CFX Duet, using the same reaction mix and template preparation. Each condition was run with ten replicates (four for 20 µL). Amplification plots, mean Cq values, and standard deviations are shown below each panel. **D.** An additional titration was performed on the Duet platform using reaction volumes from 1 to 12 µL in 1 µL increments. Each condition was tested with eight replicates. Amplification plots, mean Cq values, and standard deviations are shown. Full Cq data are available in the Supplementary Data file.

3. Reproducibility of Replicate Assays *(Figure S3)*

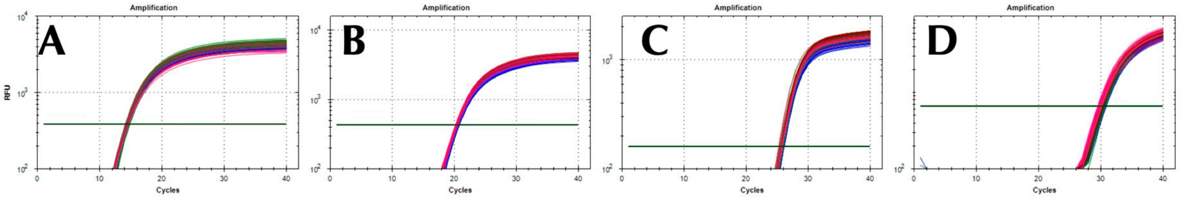

	A1	A2	A3	A4
Mean Cq	14.49	14.54	14.23	14.41
SD	0.09	0.15	0.09	0.13

	B1	B2	B3	B4
Mean Cq	20.47	20.33	20.31	20.35
SD	0.19	0.09	0.09	0.11

	C1	C2	C3	C4
Mean	25.84	25.71	25.64	25.67
SD	0.16	0.21	0.14	0.12

	D1	D2	D3	D4
Mean Cq	30.32	30.44	30.08	29.90
SD	0.21	0.18	0.24	0.27

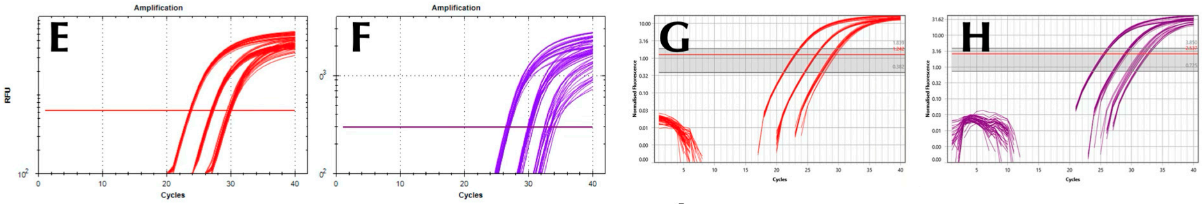

I

CDKN1

Opus	A1	A2	A3	B1	B2	B3	C1	C2	C3
Mean	23.58	23.52	23.68	27.10	27.06	27.15	29.74	29.68	29.79
SD	0.05	0.04	0.09	0.12	0.11	0.16	0.26	0.23	0.18

IGF-1

Opus	A1	A2	A3	B1	B2	B3	C1	C2	C3
Mean	26.70	26.62	26.86	30.03	30.11	30.19	32.84	34.05	33.35
SD	0.19	0.17	0.21	0.14	0.32	0.28	0.46	1.75	0.55

J

CDKN1

Mic	A1	A2	A3	B1	B2	B3	C1	C2	C3
Mean	22.94	22.94	22.94	26.34	26.41	26.41	29.08	28.95	29.27
SD	0.14	0.12	0.07	0.09	0.08	0.09	0.14	0.16	0.23

IGF-1

Mic	A1	A2	A3	B1	B2	B3	C1	C2	C3
Mean	25.54	25.41	25.62	28.94	28.91	29.10	31.43	32.03	32.48
SD	0.13	0.11	0.05	0.17	0.20	0.16	0.31	0.58	0.31

Supplementary Figure S3. Reproducibility of replicate assays across target concentrations and assays. A-D: amplification plots and Cq statistics for ACTA2-A at four input concentrations. **A.** High (~10^7 copies). **B.** Moderate (~2×10^5 copies). **C.** Low (~5×10^3 copies). **D.** Very low (~5×10^2 copies). Each concentration set consisted of four independently prepared reaction mixes (e.g., A1–A4), each run in 24 technical replicates (n = 96 per set). **E–F**. Amplification plots for CDKN1 (red, Texas Red) and IGF-1 (purple, Cy5) duplex assays at moderate, low, and very low cDNA concentrations run on the BioRad Opus. **G-H.** Amplification plots for CDKN1 (red, Texas Red) and IGF-1 (purple, Cy5) duplex assays at moderate, low, and very low cDNA concentrations run on the BMS Mic. **I-J.** Summary tables showing Cq values and descriptive statistics for CDKN1 and IGF-1 on the Opus (I) and Mic (J). On both instruments, in one reaction the CDKN1 result was normal, but the duplexed IGF-1 was an outlier. These were removed from analysis. All Cq values are listed in the supplementary data file.

4. Inter-Instrument Uniformity *(Figure S4)*

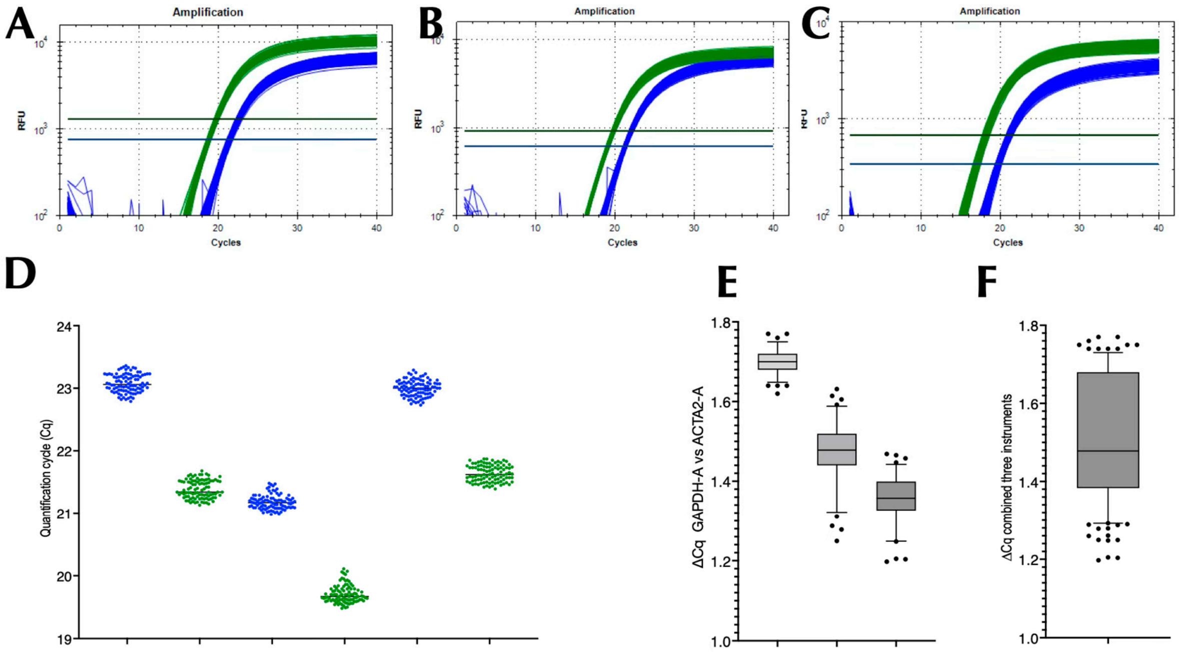

Instrument	Opus		Duet		Connect	
Target	GAPDH-A	ACTA2-A	GAPDH-A	ACTA2-A	GAPDH-A	ACTA2-A
Mean	23.08	21.38	21.18	19.71	23.00	21.64
Minimum	22.79	21.13	20.98	19.48	22.73	21.39
Maximum	23.36	21.68	21.48	20.11	23.29	21.87
Range	**0.57**	**0.55**	**0.50**	**0.63**	**0.56**	**0.48**

Instrument	Opus	Duet	Connect
Mean	**1.70**	**1.47**	**1.36**
SD	0.03	0.07	0.06
95% CI	0.01	0.01	0.01
CV	1.8%	4.7%	4.1%

ΔCq	Opus+Duet+Connect
Mean	1.51
SD	0.15
95% CI	0.02
CV	10%

Supplementary Figure S4. Inter-instrument uniformity across three 96-well Bio-Rad qPCR platforms. Amplification plots for 5µL duplex qPCR reactions detecting GAPDH-A (FAM, blue) and ACTA2-A (HEX, green). A single reaction master mix was used across all instruments. **A.** Bio-Rad Opus. **B.** Bio-Rad Duet. **C.** Bio-Rad Connect. **D.** Distribution of Cq values for GAPDH-A and ACTA2-A on each platform, including descriptive statistics (mean, SD, range, 95% CI). **E.** ΔCq (GAPDH-A – ACTA2-A) box plots per instrument, with CV% summarised in the embedded table. **F.** ΔCq distribution from pooled data across all three instruments. Whiskers represent the 95% confidence interval. All reactions were run in 96 replicates. All Cq values are listed in the supplementary data file.

5. Correlation with expected copy numbers ***(Figure S5)***

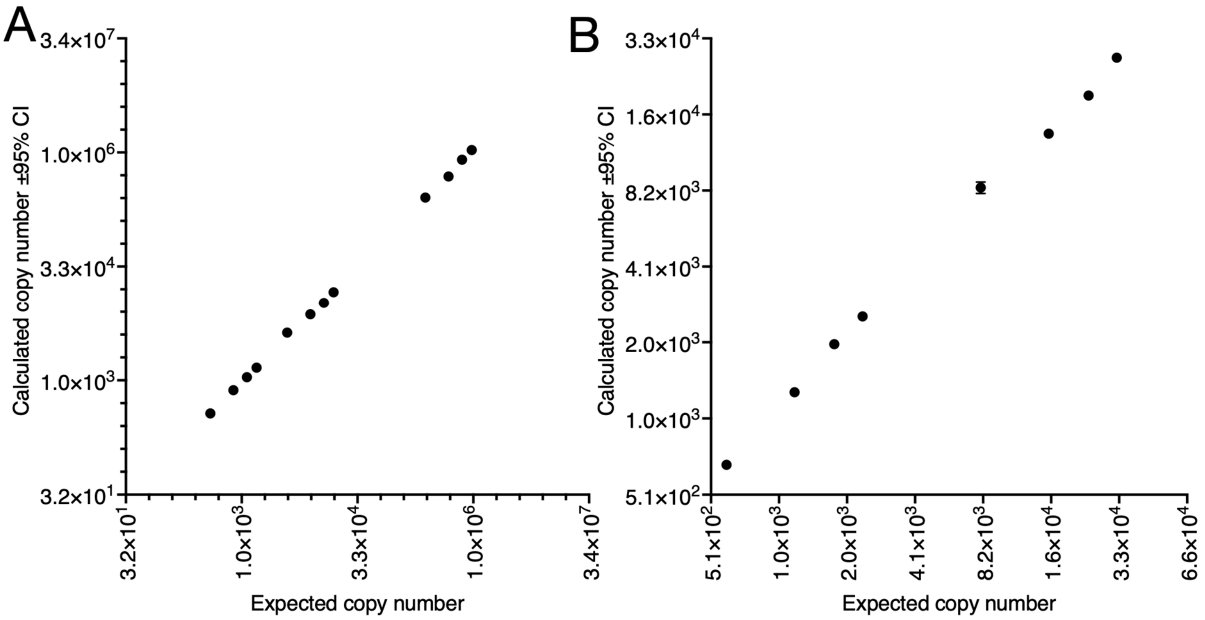

Supplementary Figure S5. Log$_2$-transformed expected versus measured copy numbers. A. Bio-Rad Opus platform. **B.** Mic instrument. Each point represents the mean calculated copy number from 12 technical replicates per sample, plotted against the expected input concentration. Error bars denote 95% confidence intervals. Data show tight agreement with expected values across more than three orders of magnitude. Increased variability at lower copy numbers is reflected in wider confidence intervals, particularly on the Mic platform.

.

6. Alternative duplex can increase variability *(Figure S6)*

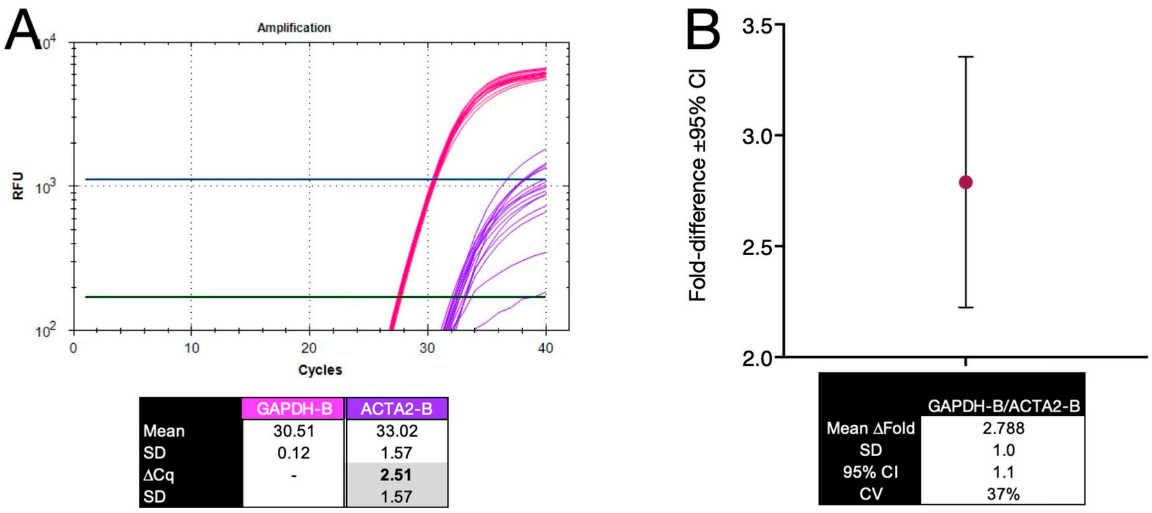

	GAPDH-B	ACTA2-B
Mean	30.51	33.02
SD	0.12	1.57
ΔCq	-	**2.51**
SD		1.57

	GAPDH-B/ACTA2-B
Mean ΔFold	2.788
SD	1.0
95% CI	1.1
CV	37%

Supplementary Figure S6. Variability in fold-change quantification using an alternative duplex assay (GAPDH-B/ACTA2-B). A. Amplification plots and descriptive statistics for GAPDH-B (pink) and ACTA2-B (purple) in duplex reactions performed at very low target concentrations. ACTA2-B amplification exhibited increased variability relative to GAPDH-B, as reflected in the higher standard deviation and broader ΔCq distribution. B. Calculated fold-difference (GAPDH-B normalised to ACTA2-B) with associated 95% confidence interval. The large interval and high coefficient of variation (37%) contrast with the narrower intervals observed in the primary duplex assays (Figure 5H), indicating reduced reproducibility. These data highlight the importance of primer/probe design and potential duplex interaction effects on relative quantification accuracy. All Cq values are listed in the supplementary data file.

7. Multiplex relative quantification *(Figure S7)*

A

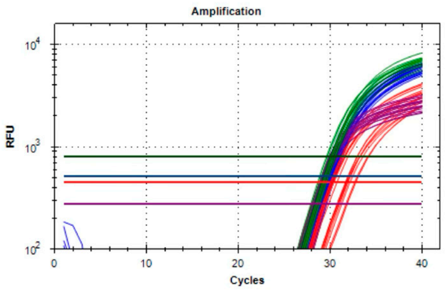

B

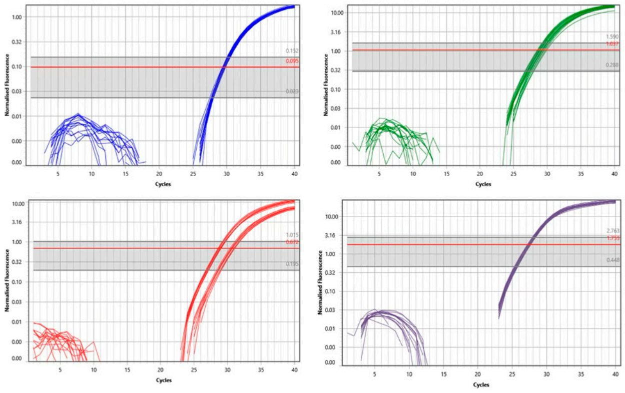

	Sample 1				Sample 2			
	CDKN1	IGF-1	GAPDH-A	ACTA2-A	CDKN1	IGF-1	GAPDH-A	ACTA2-A
Mean	32.03	28.12	30.13	30.24	30.21	28.39	30.10	30.12
SD	0.43	0.12	0.25	0.18	0.13	0.17	0.24	0.38

	Sample 1				Sample 2			
	CDKN1	IGF-1	GAPDH-A	ACTA2-A	CDKN1	IGF-1	GAPDH-A	ACTA2-A
Mean	31.04	27.27	29.52	29.22	28.85	27.42	29.47	28.72
SD	0.20	0.08	0.26	0.39	0.18	0.16	0.12	0.26

C

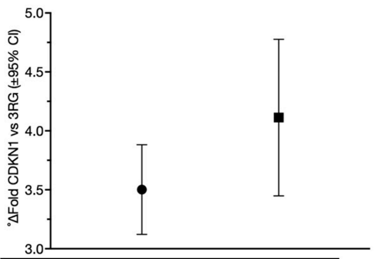

Instrument	Opus	Mic
Mean ΔFold	3.50	4.10
SD	0.45	0.79
95% CI	0.38	0.66
CV	0.13	0.19

Supplementary Figure S7. Accuracy of fold-difference quantification in multiplex qPCR. Two samples containing the same concentrations of IGF-1, GAPDH-A and ACTA2-A, but 4x that of CDKN1 were amplified using the Bio-Rad Opus and BMS Mic. **A.** Amplification plots and descriptive statistics recorded by the Bio-Rad Opus. **B.** Amplification plots and descriptive statistics recorded by the BMS Mic. The Mic software does not permit the plotting of the FAM and HEX amplification curves on the same plot. **C.** Fold-difference in concentration (±95% CI) in sample 2 relative to sample 1 for CDKN1 normalised against the geometric mean of GAPDH, CDKN1 and ACTA2-A. Whiskers indicate the upper and lower bounds of the 95% CI. All quantification cycle (Cq) values are listed in the supplementary data file.
